# Nitration-driven structural changes in Hsp90 linked to gain of pathological functions

**DOI:** 10.1042/BCJ20253230

**Published:** 2025-08-20

**Authors:** Tilottama Chatterjee, Alfonso Taboada, Isabelle E. Logan, Patience N. Paul, Miranda Huerta, Patrick Reardon, Rafael Radi, Ari Zeida, Maria Clara Franco

**Affiliations:** 1 Department of Biochemistry and Biophysics, Oregon State University, Corvallis, OR 97331, U.S.A; 2 Departamento de Métodos Cuantitativos, Facultad de Medicina, Universidad de la República, Montevideo, CP, 11800, Uruguay; 3 Departamento de Bioquímica, Facultad de Medicina, Universidad de la República, Montevideo, CP, 11800, Uruguay; 4 Centro de Investigaciones Biomédicas (CEINBIO), Facultad de Medicina, Universidad de la República, Montevideo, CP, 11800, Uruguay; 5 Center for Translational Science, Florida International University, Port St. Lucie, FL, 34987, U.S.A; 6 Department of Cellular and Molecular Medicine, Herbert Wertheim College of Medicine, Florida International University, Miami, FL, 33199, U.S.A

**Keywords:** heat shock protein 90 (Hsp90), peroxynitrite, post-translational modification (PTM), reactive nitrogen species (RNS), structure-function, tyrosine nitration

## Abstract

Protein tyrosine (Y) nitration is an oxidative modification that occurs in pathological conditions such as neurodegenerative diseases and solid tumors. Depending on the location of the tyrosine residue, nitration can modify protein structure and function and affect cellular processes. We previously showed that site-specific nitration of the molecular chaperone heat shock protein 90 (Hsp90) leads to distinct pathological gain-of-function that cannot be compensated or overcome by native Hsp90. While Hsp90 nitrated on Y33 localizes in mitochondria and decreases mitochondrial metabolism, Hsp90 nitrated on Y56 activates the purinergic receptor and calcium channel P2X7, triggering downstream signaling pathways that can lead to either cell proliferation or apoptosis, depending on the cell type. Herein, using complementary biophysical, biochemical, and *in silico* methods, we show that nitration on Y33 and Y56 triggers significant site-dependent local and global structural changes linked to changes in Hsp90 activity. Nitration of these critical residues led to destabilization of Hsp90 dimer and formation of stable oligomeric species, with differential effects on Hsp90 ATPase and chaperone holdase activities depending on the nitrated residue. Molecular dynamics simulations further support the impact of nitration on Y33 and Y56 on the ATP-lid dynamics and the interaction of ATP with R392, critical to Hsp90 ATPase activity. Establishing the molecular basis of nitration-induced structural changes in Hsp90 leading to disease-driving functions is the first step toward the development of therapeutic approaches selectively targeting these pathological variants of Hsp90.

## Introduction

The production of reactive oxygen and nitrogen species (ROS and RNS, respectively) in pathologies such as neurodegeneration and cancer is well established [1–4]. While ROS and RNS have been long viewed as nonspecific and largely damaging species, a novel role as disease drivers that can cause selective modifications with well-defined downstream signaling effects has started to emerge [5]. Among the RNS, peroxynitrite is a potent oxidant produced by cells in pathological conditions, generated from the reaction between nitric oxide and superoxide [6,7]. In the cell, the radical products of peroxynitrite decomposition react with tyrosine residues in proteins to form 3-nitrotyrosine (3-NY) [3]. NY is a known biomarker for the presence of oxidative stress in multiple pathologies [8–10]. However, whether NY was just a by-product of the oxidative environment or an actual player in the disease process was difficult to determine due to the multiplicity of potential targets and oxidative modifications to proteins introduced by peroxynitrite [11,12]. The presence of peroxynitrite and NY has been associated with the changes in the functions of multiple proteins; manganese-superoxide dismutase is inactivated by the nitration of a single tyrosine residue [13]; nitration of calmodulin alters its sensitivity for calcium [14], while nitration of fibrinogen accelerates its ability to form fibrin [15]. In acute lung injury and pulmonary hypertension, nitration activates the small GTPase RhoA, leading to dysregulation of metabolic and signaling pathways [16–20], and in the case of cytochrome *c*, nitration promotes a gain-of-peroxidatic function and nuclear translocation [21,22]. The combined identification over the last 30 years of a nitroproteome comprising nearly 1000 proteins that participate in critical cellular processes and undergo tyrosine nitration [11,23,24] could have profound implications for cellular signaling. Supporting a regulatory role for nitrated proteins in pathological conditions, we previously showed that nitration of heat shock protein 90 (Hsp90) induces a distinct pathogenic gain-of-function depending on the tyrosine (Y) that is nitrated [25–28].

The molecular chaperone Hsp90 is a ubiquitous cytosolic protein expressed in all eukaryotic cells [29,30]. Hsp90 levels account for 1–2% of total cytosolic protein under normal physiological conditions and up to 4–6% under stress conditions [31,32]. Two cytosolic isoforms of Hsp90 are expressed in mammalian cells, the constitutively expressed Hsp90β and the inducible Hsp90α [29]. The primary role of Hsp90 *in vivo* is chaperoning a vast array of client proteins, facilitating folding, remodeling, and/or maturation [33,34]. To date, Hsp90 has over 300 known client proteins involved in multiple cellular processes, including kinases, transcription factors, and pro-survival and pro-apoptotic proteins [29,32,35,36]. For many clients, Hsp90 chaperone activity is supported by ATP binding to a pocket in N-terminal nucleotide-binding domain (Hsp90-NTD), followed by ATP hydrolysis [37]. Human Hsp90β (UniProtKB accession number P08238, gene HSP90AB1) contains 24 tyrosine residues, of which 5 are susceptible to nitration after treatment of the protein with peroxynitrite *in vitro* (Y33, Y56, Y276, Y484, and Y596) [27]. We showed that nitration on two critical residues, Y33 and Y56, induces distinct pathological functions in Hsp90. Hsp90 nitrated at Y56 binds to and activates the purinergic P2 × 7 receptor in motor neurons and PC12 cells, which triggers death in both cell types by activating distinct signaling pathways [27,28]. On the other hand, while nitration at Y33 triggers motor neuron death [27], in PC12 cells, the nitrated protein does not induce cell death [28] instead forms a mitochondrial complex that decreases mitochondrial activity [25]. Further, we found that nitrated Hsp90 leads to schwannoma cell proliferation by acting as a metabolic switch [38]. While Hsp90 nitrated at Y33 decreases mitochondrial activity, Hsp90 nitrated at Y56 increases glycolysis through the activation of P2×7 receptor [38]. We developed selective monoclonal antibodies against Hsp90 nitrated on Y33 or Y56 and showed that the chaperone is endogenously nitrated on these tyrosine residues *in vivo* in motor neurons from amyotrophic lateral sclerosis patients and animal models [27], in PC12 cells treated with peroxynitrite [27,28], and in human schwannomas and cell culture models [26]. Collectively, these results suggest that site-specific nitration induces structural changes in Hsp90 that trigger distinct pathological functions that unmodified Hsp90 does not normally perform.

**Figure 1:**
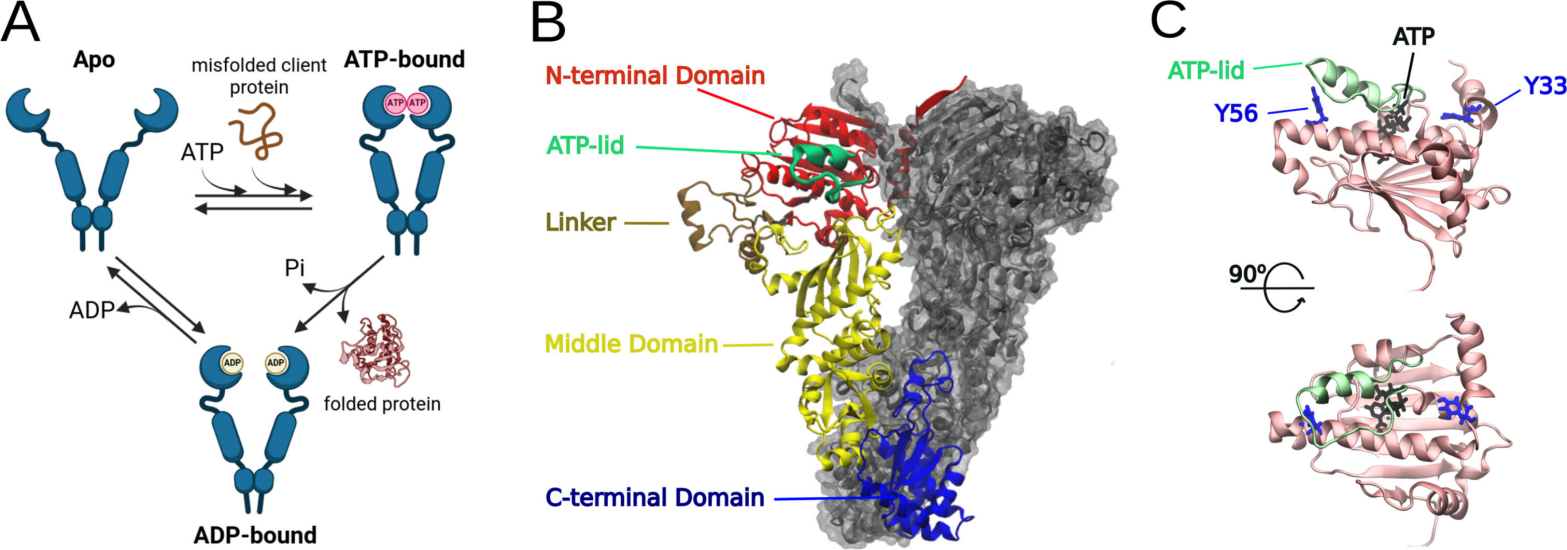
Catalytic mechanism and structure of Hsp90β. (**A**) Simplified cycle of Hsp90β conformational changes during ATP hydrolysis and binding to client protein. (**B**) Structure of the Hsp90β dimer in the closed conformation, highlighting the N- (Hsp90-NTD), middle- (Hsp90-MD), C-terminal (CTD) domains, along with the ATP-lid and linker regions. (**C**) Location of Y33 and Y56 with respect to the ATP-binding pocket in the Hsp90-NTD.

Hsp90 function relies on stable dimer formation. Each protomer includes three distinct domains, the Hsp90-NTD, the client-binding middle domain (Hsp90-MD), and the C-terminal dimerization domain (CTD) [39]. Apo-Hsp90 is constitutively dimerized through the CTD and exists in a conformation that resembles a ‘flying seagull’, adopting a range of conformations between a completely extended state and a closed state [40,41] (Figure 1A–B). The closed conformation is stabilized by ATP binding to the N-terminal nucleotide-binding pocket, which drives the NTDs of the two protomers to interact, as well as conformational changes in a segment of the NTD that forms a lid over the ATP-binding site [42]. This is followed by subsequent ATP hydrolysis and release of ADP [29,43] (Figure 1A). Hsp90 chaperone activity partially relies on ATP binding and hydrolysis [37,44,45]. Nitration at Y33 and/or Y56, both located in Hsp90-NTD (Figure 1C), confers distinct site-dependent disease-driving functions [25–28]. To understand the nature of the different biological functions conferred by nitration of each individual residue, here, we further dissected the role of site-specific nitration of Y33 *versus* Y56 on Hsp90 structure and activity and probed into modeling the intramolecular interactions. Nitration of Hsp90 at Y33 and/or Y56 significantly affects the local environment of both Y33 and Y56 and triggers global structural changes, leading to destabilization of Hsp90 dimeric state and formation of higher-order structures with associated changes in activity. This is the first comprehensive characterization of protein tyrosine nitration-induced structural changes leading to distinct novel pathogenic functions.

## Results

### Nitration at Y33 and/or Y56 induces significant changes in Hsp90 secondary and quaternary structure

**Figure 2:**
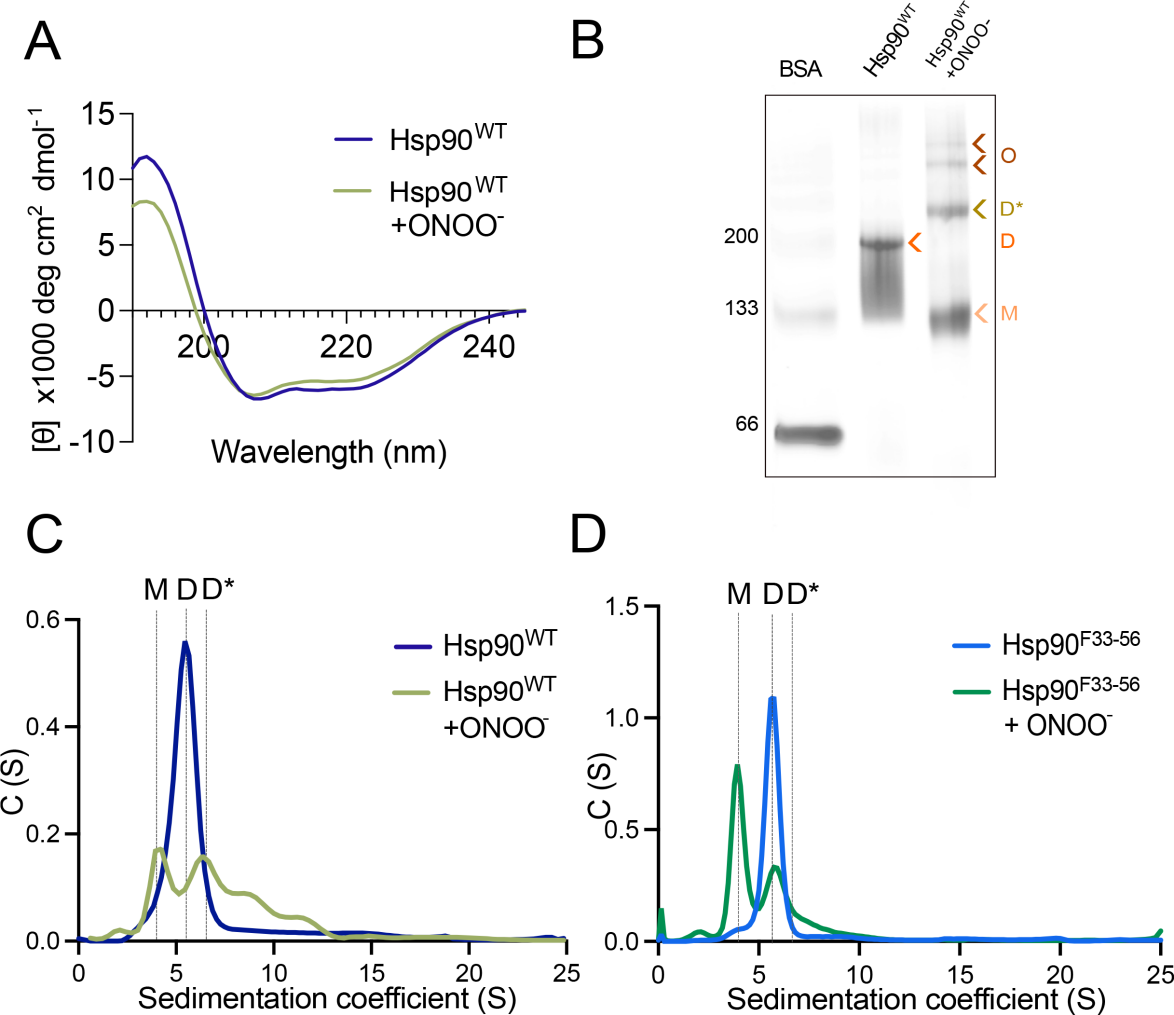
Peroxynitrite treatment alters Hsp90 secondary and quaternary structures. (**A**) Hsp90 secondary structure (11.6 μM monomer) with and without treatment with 0.5 mM peroxynitrite (ONOO^-^) was assessed by circular dichroism (CD). (**B**) Effects of peroxynitrite treatment on Hsp90 quaternary structure were assessed by native polyacrylamide gel electrophoresis. (**C-D**) Sedimentation profile of Hsp90 and peroxynitrite-treated Hsp90 (**C**), and Hsp90 in which Y33 and Y56 were replaced by nitration-resistant phenylalanine, treated or not with peroxynitrite (**D**) after performing analytical ultracentrifugation (AUC) sedimentation velocity. C(S), sedimentation coefficient distribution; D, dimer, open conformation; D*, dimer, closed conformation; M, monomer; O, oligomers.

We showed that the nitration of Hsp90 at Y33 and Y56 leads to distinct pathological functions. On the one hand, nitration at Y33 decreases mitochondrial activity in PC12 and schwannoma cells [28,38], while nitration at Y56 leads to activation of P2×7R, which induces motor neuron and PC12 cell death [27,28] but increases glycolysis and schwannoma cell proliferation [38]. Collectively, these results suggest that nitration triggers differential structural changes in Hsp90 that are site-specific and lead to these new pathological functions. We first assessed the impact of peroxynitrite treatment on recombinant human Hsp90β (Hsp90^WT^) secondary structure using Far-UV circular dichroism (CD). Treatment of Hsp90^WT^ with 0.5 mM peroxynitrite, a concentration that induces the pathological functions, and the nitration of Y33, Y56, Y276, Y484, and Y596 [27] resulted in a loss of helical content observed through reduced positive ellipticity at 190 nm, together with reduced negative ellipticity at 202 and 222 nm (Figure 2A). The Hsp90 dimer exists in a series of conformational states through the interaction of residues located in the Hsp90-NTD and Hsp90-MD [42,46–48]; thus, it is possible that the peroxynitrite-induced changes observed in Hsp90 secondary structure could translate into changes in its quaternary structure or originate from the destabilization of Hsp90 dimer. We confirmed the prevalence of a single 180 kDa dimeric population in Hsp90^WT^ by native polyacrylamide gel electrophoresis (PAGE) (Figure 2B). Further, we observed the formation of higher molecular weight species (Figure 2B), suggesting oxidation by peroxynitrite induced the formation of Hsp90 oligomers.

To determine the populations of Hsp90 present before and after peroxynitrite treatment, we performed sedimental velocity analytical ultracentrifugation (AUC). As shown in Figure 2C, Hsp90^WT^ presented as a single population with a peak at sedimentation coefficient (S) of 5.6, as reported previously [49,50]. On the other hand, peroxynitrite-treated Hsp90^WT^ showed a decrease in the dimer population and the presence of two additional peaks, one at a lower sedimentation coefficient (3.9S) corresponding to a monomeric form and two peaks at higher sedimentation coefficients (8.6S and 11.6S), indicative of higher-order oligomeric species. The 11S population corresponds to the expected sedimentation coefficient for a tetramer [49], while a peak at 8.6S has not been previously observed for Hsp90 but may indicate presence of a trimer. Furthermore, we observed a slight shift in the sedimentation coefficient of the dimer (5.6 S to 6.4 S, Table 1), in agreement with a previous report showing a similar shift for a dimer adopting a closed conformation [50]. The formation of the oligomeric species and the shift in the sedimentation coefficient of the dimer were, at least in part, due to nitration at Y33 and/or Y56, as no peroxynitrite-induced oligomer formation was observed after replacing Y33 and Y56 by nitration-resistant phenylalanine (Hsp90^F33-56^), and the dimer presented as a 5.6S peak, similar to untreated Hsp90^WT^ and Hsp90^F33-56^ (Figure 2D). The presence of a peak corresponding to monomeric species in Hsp90^F33-56^ after peroxynitrite treatment indicates that, as opposed to the oligomeric species, the formation of monomers may be driven by oxidation of amino acids other than Y33 and Y56, such as Y276, Y484, and Y596 that we showed are also prone to nitration. Collectively, these results indicate that nitration at Y33 and Y56 induces significant structural changes, affecting Hsp90 quaternary structure.

**Table 1:** Sedimentation coefficients [S] corresponding to Hsp90 and different populations of nitrated Hsp90.

Protein	Monomer [S]	Dimer [S]	Oligomer [S]
Hsp90^WT^	-	5.7 ± 0.1	-
Hsp90^WT^ + ONOO^-^	4.1 ± 0.2	6.5 ± 0.1	8.6 ± 0.1
			11.6 ± 0.2
Hsp90^F33-56^	-	5.7 ± 0.1	-
Hsp90^F33-56^ + ONOO^-^	4.0 ± 0.1	5.7 ± 0.1	-
Hsp90^NY33^	4.1 ± 0.1	5.6 ± 0.2	14.1 ± 0.3
Hsp90^NY56^	-	5.6 ± 0.2	14.2 ± 0.1
Hsp90^NY33-56^	-	6.1 ± 0.2	14.6 ± 0.2

The sedimentation coefficients of wildtype Hsp90 (Hsp90^WT^), Hsp90 in which Y33 and Y56 were replaced by nitration-resistant phenylalanine (Hsp90^F33-56^), peroxynitrite-treated Hsp90 or Hsp90^F33-56^ (+ ONOO^-^), and the different site-specific forms of nitrated Hsp90 were determined by analytical ultracentrifugation sedimentation velocity.

**Figure 3:**
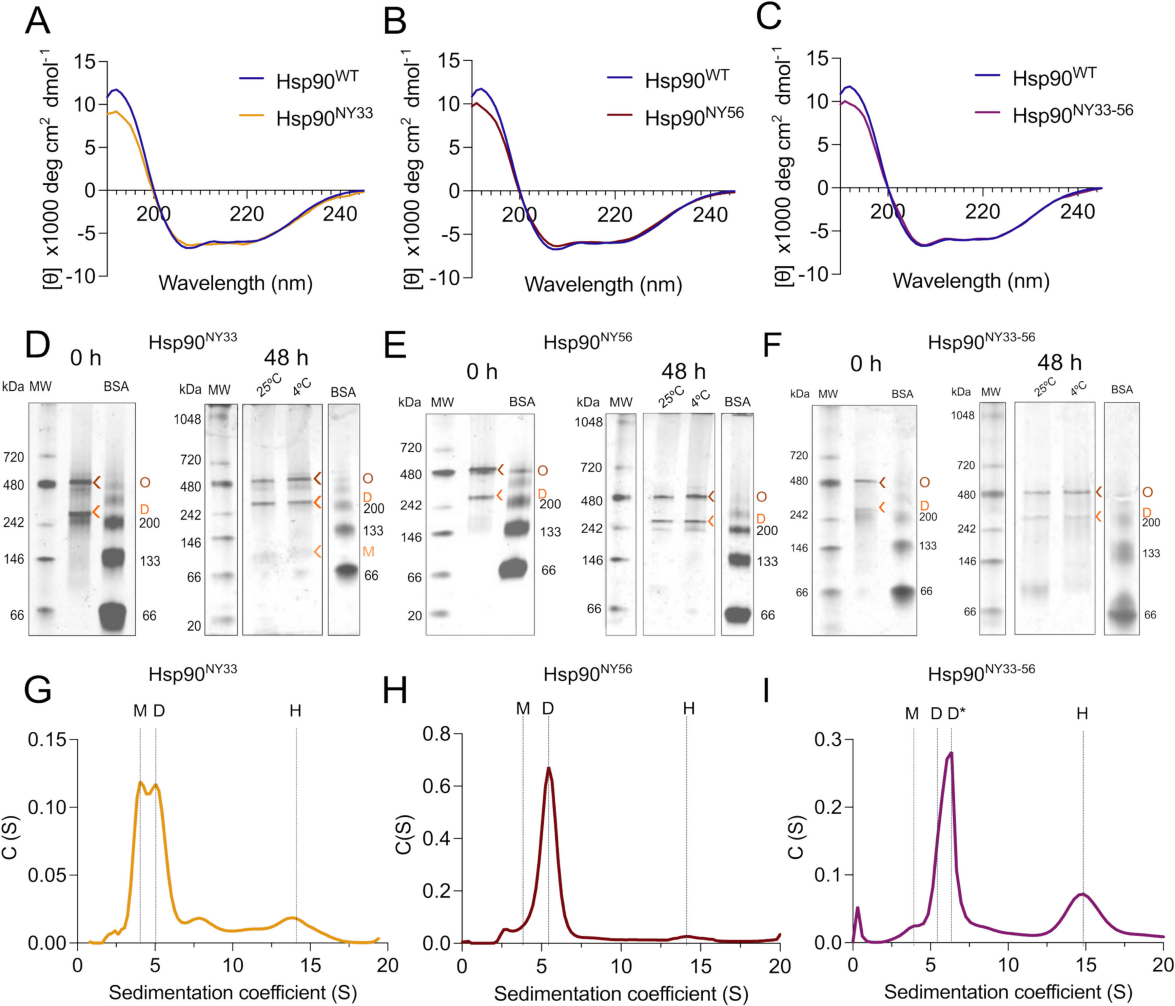
Nitration at Y33 and Y56 triggers structural changes in Hsp90. (**A-C**) Changes in Hsp90 secondary structure after site-specific incorporation of nitrotyrosine at (**A**) position 33 (Hsp90^NY33^), (**B**) position 56 (Hsp90^NY56^), and (**C**) both positions simultaneously (Hsp90^NY33-56^) were investigated using circular dichroism. (**D-E**) The effect of site-specific nitration at (**D**) Y33, (**E**) Y56, and (**F**) simultaneous nitration at Y33 and Y56 on Hsp90 quaternary structure was assessed by native polyacrylamide gel electrophoresis (PAGE) immediately after purification, and after 48 h incubation at 4°C and 25°C. D, dimer; M, monomer; O, oligomers. (**G-I**) Analytical ultracentrifugation (AUC) sedimentation velocity profiles of (**G**) Hsp90^NY33^, (**H**) Hsp90^NY56^, and (**I**) Hsp90^NY33-56^ show the sedimentation coefficient (**S**) *versus* the sedimentation coefficient distributions c(s). D, dimer, open conformation; D*, dimer, closed conformation; H, hexamer; M, monomer; S, sedimentation coefficient.

To dissect the site-specific contributions of nitration at Y33 and Y56 on Hsp90 structural changes, we produced recombinant Hsp90 carrying a single NY at one or both specific positions as the sole oxidative modification in the protein using genetic code expansion [25,27]. First, we assessed the impact of nitration at Y33 and Y56 on Hsp90 secondary structure by CD analysis. Hsp90^NY33^ showed a decrease in the absorbance peak at 195 nm and bridging of the peaks at 202 and 222 nm, indicating a shift from alpha helix to beta sheet presence in the secondary structure composition (Figure 3A and Table 1). In contrast, the presence of NY at position 56 led to a decrease in the peak at 195 nm, indicating loss of helical structure in Hsp90^NY56^ (Figure 3B and Table 2). Surprisingly, the simultaneous nitration of Y33 and Y56 did not show an additive effect. This protein only showed minor loss of α-helical content (Figure 3C and Table 1).

**Table 2:** Secondary structure composition of wild-type (WT) and nitrated forms of Hsp90.

Sample	Alpha helix	Beta sheet
Hsp90^WT^	33.8 ± 0.3%	27.6 ± 1.1%
Hsp90^NY33^	23.6 ± 0.5%	33.1 ± 1.7%
Hsp90^NY56^	31.1 ± 0.5%	28.3 ± 0.5%
Hsp90^NY33-56^	32.0 ± 0.6%	27.9 ± 2.3%

**Figure 4:**
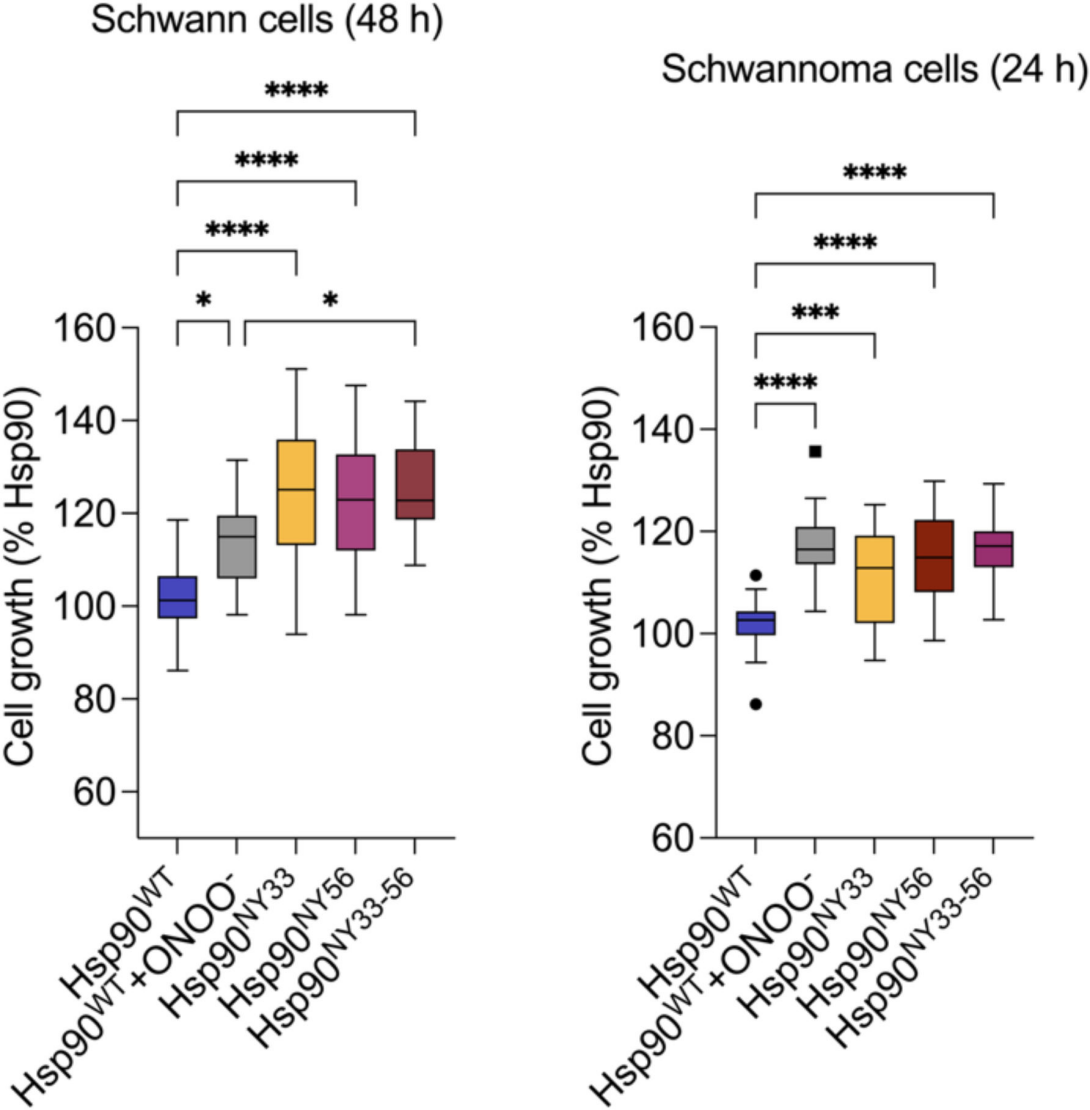
Simultaneous nitration of Hsp90 at Y33 and Y56 increases schwannoma and Schwann cell proliferation. Human recombinant Hsp90^WT^, Hsp90^WT^ treated with 0.5 mM peroxynitrite, Hsp90^NY33^, Hsp90^NY56^, and Hsp90^NY33-56^ were intracellularly delivered to human Schwann cells or mouse schwannoma cells, and cell growth assessed at 48 and 24 h, respectively. Data are shown using Tukey’s representation with the median indicated in the box plot (*n* = 3–4 independent experiments). ****P<0.0001, *P<0.05 *versus* Hsp90^WT^ by one-way ANOVA with Dunnett’s multiple comparisons *post hoc* test.

**Figure 5:**
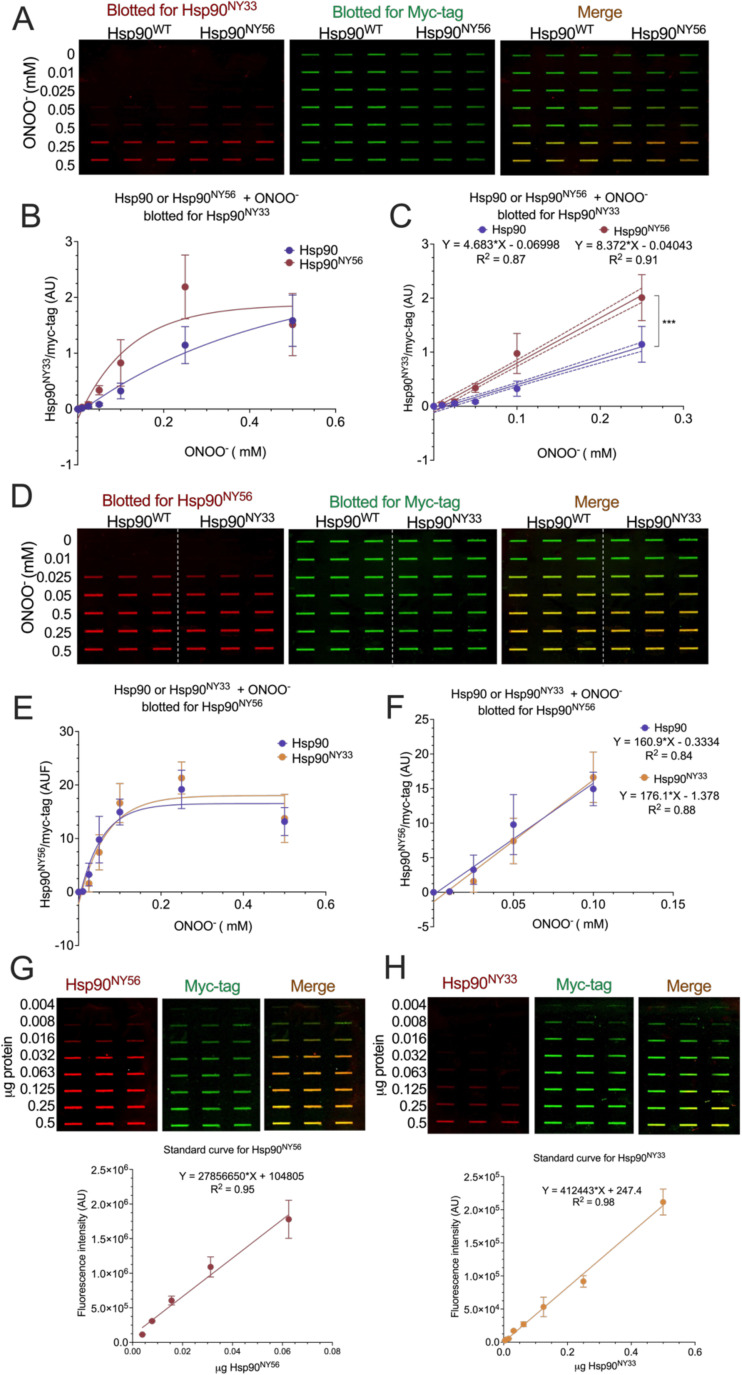
Nitration of Hsp90 at Y56 facilitates nitration at Y33. Hsp90^WT^, Hsp90^NY33^, or Hsp90^NY56^ were treated with increasing concentrations of peroxynitrite, as indicated (0–0.5 mM), and nitration at either (**A-C**) Y56 (for Hsp90^NY33^ + ONOO^-^) or (**D-F**) Y33 (for Hsp90^NY56^ + ONOO-) assessed by slot blot *versus* Hsp90^WT^ + ONOO^-^ using monoclonal antibodies against either Hsp90^NY33^ or Hsp90^NY56^ (red), and normalized for total Hsp90 using an anti-myc antibody (green). (**A**) Slot blot for Hsp90^WT^ and Hsp90^NY56^ blotted for Hsp90^NY33^. (**B**) Quantitation of the infrared signals (Hsp90^WT^ or Hsp90^NY56^/myc) in the full peroxynitrite concentration range (0–0.5 mM), and (**C**) in the liner range of the curves (0–0.25 mM) with corresponding regressions. (**D**) Slot blot for Hsp90^WT^ and Hsp90^NY33^ blotted for Hsp90^NY56^. (**E**) Quantitation of the infrared signals (Hsp90^NY56^ or Hsp90^NY33^/myc) in the full peroxynitrite concentration range (0–0.5 mM), and (**F**) in the liner range of the curves (0–0.1 mM) with corresponding regressions. (**G-H**) Standard curves for the antibodies against (**G**) Hsp90^NY33^ or (**H**) Hsp90^NY56^ showing that the antibody against Hsp90^NY56^ presents an order of magnitude higher affinity for Hsp90^NY56^ than the antibody against Hsp90^NY33^ for Hsp90^NY33^. Merge indicates the overlap of the red and green signals. graphs represent the mean ± SD (*n* = 3 independent experiments), ***P<0.0001 by *F*-test.

The analysis of nitration-induced changes in Hsp90 quaternary structure revealed the presence of monomer, dimer, and a band corresponding to an oligomeric species in Hsp90^NY33^ by native PAGE (Figure 3D). Furthermore, Hsp90^NY56^ also showed the presence of an oligomeric peak, in addition to the dimer present at ~180 kDa in the native gels, as evidenced by the presence of a band at a higher molecular weight compared with the dimer (Figure 3E). In contrast, although the simultaneous nitration of Y33 and Y56 induced minor changes in secondary structure, it affected Hsp90 quaternary structure, triggering distinct structural changes. In native gels and as observed after incorporation of a single NY, Hsp90^NY33-56^ showed two discrete bands at 180 and 540 kDa (Figure 3F). For all three nitrated proteins, the dimer and oligomeric populations were stable at 4°C and 25°C for at least 48 h (Figure 3D–F) and disassembled in denaturing conditions, as evidenced by the presence of a band of ~90 kDa apparent molecular weight corresponding to the monomeric species (Supplementary Figure 1). The stability of the different populations at 25°C enabled the use of complementary methodologies such as AUC that requires long centrifugation times at room temperature (~12–15 h) to assess the effect of nitration on Y33 and/or Y56 on Hsp90 structure. In agreement with the previous results, Hsp90^NY33^ sedimentation profile showed two overlapping peaks at sedimentation coefficients of 4.1 and 5.6, suggesting the presence of an unstable dimeric population undergoing rapid exchange with monomeric species, and a third peak at 14.1, corresponding to the oligomeric species (Figure 3G and Table 1). In contrast, the Hsp90^NY56^ sedimentation profile showed a predominant peak corresponding to the dimer (5.6S), similar to Hsp90^WT^ profile (Figure 3H). Unlike Hsp90^NY33^, the presence of a monomeric peak was not evident in Hsp90^NY56^ sedimentation profile, suggesting that nitration at Y56 does not destabilize the Hsp90 dimer, and the oligomeric peak represented a minor population. Interestingly, similar to peroxynitrite-treated Hsp90, Hsp90^NY33-56^ showed a shift in the sedimentation coefficient of the dimer, from ~5.6S to 6.1S, in agreement with the reported sedimentation coefficient of a closed Hsp90 dimer [50], suggesting that nitration at both Y33 and Y56 may induce a switch from the open to the closed conformation, in the absence of ATP. In addition, a well-defined hexamer peak at 14.6S was present (Figure 3I). These are impactful structural changes in Hsp90 triggered by nitration at Y33 and/or Y56. A question that remains unanswered is whether simultaneous Hsp90 nitration at Y33 and Y56 triggers a proliferative activity, as we showed for Hsp90^NY33^ and Hsp90^NY56^ [38]. We confirmed that the intracellular delivery of Hsp90^NY33-56^ to either human Schwann cells or mouse schwannoma cells significantly increased cell proliferation 24 and 48 h after delivery, respectively (Figure 4), and we previously showed that the delivered recombinant proteins are still present in the cells up to 48 h after delivery [38]. We also showed that in three-dimensional schwannoma cell culture models, Hsp90^NY56^ is homogenously distributed throughout the cellular structure, but surprisingly, Hsp90^NY33^ is predominantly enriched in the outer cellular layer [38]. These results raise the possibility of those cells containing an Hsp90 subpopulation that is simultaneously nitrated on both residues. However, in the cellular context, nitration of Hsp90 at both Y33 and Y56 may not be feasible at the endogenous peroxynitrite levels produced by tumor cells. To better understand the dynamics of nitration at both Y33 and Y56, we treated either Hsp90^WT^, Hsp90^NY33^, or Hsp90^NY56^ with increasing concentrations of peroxynitrite and evaluated nitration of the other Y residue by infrared slot blot. The results indicate that the presence of NY at position 56 facilitated nitration on Y33, as nitration at Y33 in Hsp90^NY56^, was detected at lower peroxynitrite concentrations than those observed for Hsp90^WT^ (Figure 5A and B), showing an order of magnitude increase in nitration versus Hsp90^WT^ (Figure 5C). In contrast, the presence of NY at position 33 had no effect on Hsp90 nitration at Y56 *versus *Hsp90^WT^ (Figure 5D–F). Because the slot blots blotted with the antibody against Hsp90^NY56^ showed increased overall fluorescence signal when compared with the antibody against Hsp90^NY33^, which could indicate that Y56 is more susceptible to nitration than Y33, we also performed standard curves for both antibodies. The standard curves confirmed that rather than increased nitration at Y56, the antibody against Hsp90^NY56^ has an order of magnitude higher affinity for the corresponding nitrated protein than the antibody raised against Hsp90^NY33^ (Figure 5G–H). Collectively, these results suggest that Hsp90 nitration at Y33 and/or Y56 induces distinct structural changes linked to a proliferative function and that by facilitating nitration at Y33 and depending on the conditions, nitration at Y56 could lead to a fraction of Hsp90 carrying NY at both sites.

### Nitration at Y33 and Y56 affects Hsp90 intramolecular interactions

**Figure 6:**
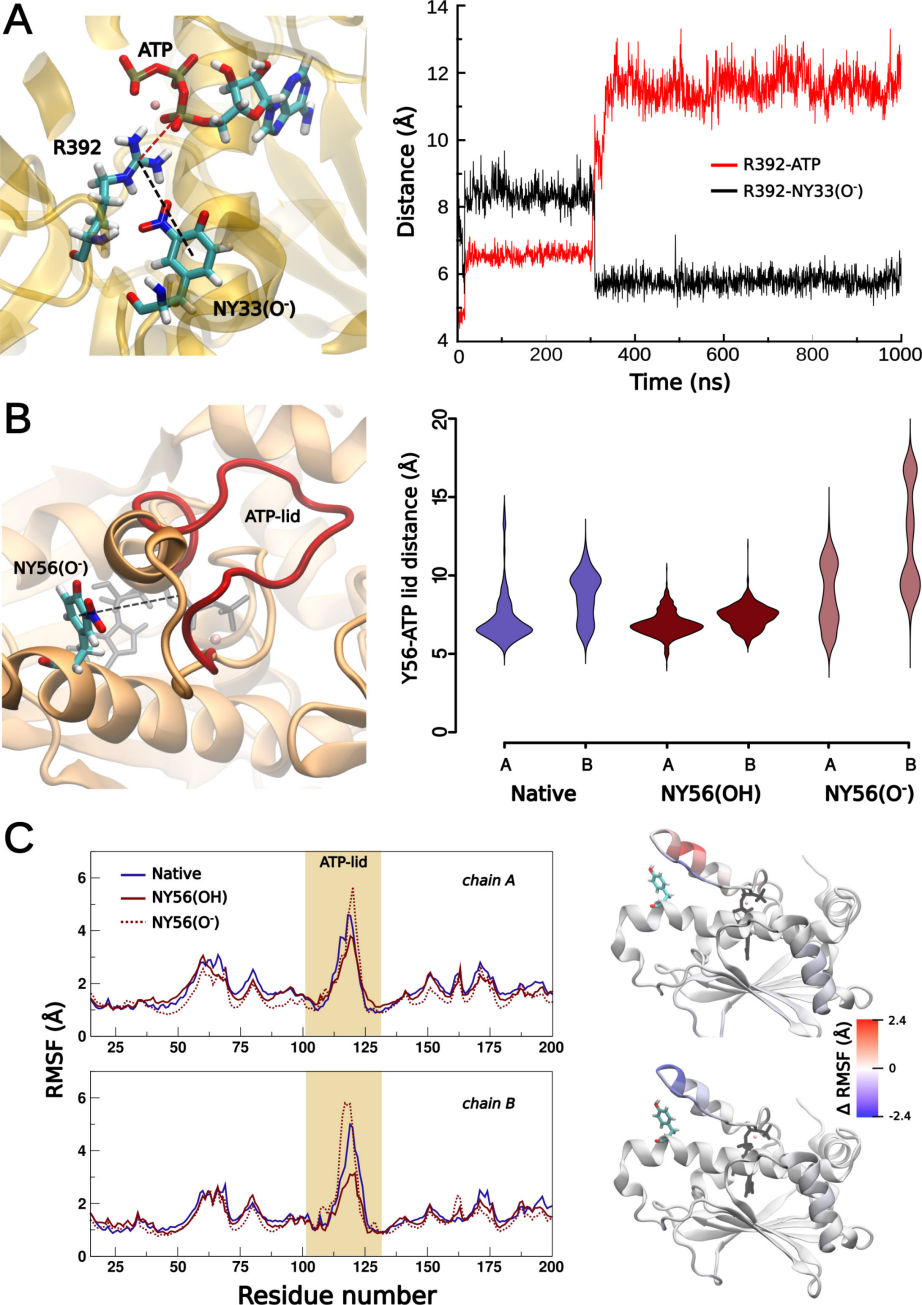
Nitration of Y33 or Y56 affects intramolecular interactions of these residues with Hsp90 middle domain and ATP-lid. (**A**) Structural representation of the interactions between Y33, R392, and ATP (left). On the right, evolution of R392 guanidinium carbon atom and ATP α-phosphorous atom distance (red trace), or R392 guanidinium carbon atom and NY33 in the deprotonated state (black trace) obtained from full-length closed homodimer molecular dynamics of Hsp90. (**B**) Interactions of NY56 with the ATP-lid. The open conformation of the ATP-lid favored by the deprotonated NY56 is superimposed and highlighted in red. Right: Y56-ATP-lid distance distributions (Å, see left) as a function of Y56 states (unmodified, nitrated and protonated, or nitrated and deprotonated) for each protomer (chains A and B) during full-length Hsp90 dimer molecular dynamics analysis. (**C**) Root mean square fluctuations (RMSF, Å) of the NTD residues as a function of Y56 state, highlighting the ATP-lid region. The differential RMSF between NY56(O^-^) and native (up) or protonated -NY56(OH)- and native (down) are mapped on the NTD structure.

To better understand the intermolecular interactions of Hsp90 residues responsible for the structural and functional changes due to nitration at Y33 and Y56, we performed molecular dynamics (MD) simulations of different Hsp90 models. As nitration affects the pKa of a tyrosine residue [51], we considered the different protonation states of each nitrated tyrosine residue. Y33 is located in a solvent-inaccessible region of the Hsp90-NTD, forming part of an interface between the N-terminal and middle domains of the two protomers (Figure 1C). Thus, we first studied the effect of nitration at Y33 on protein structure and dynamics in the closed full-length Hsp90β homodimer model. Starting from this conformation, we observed during the first steps of the MDs that Y33 interacted with R392 located in the middle domain and that this interaction was particularly strengthened by Y33 nitration in the deprotonated form (Figure 6A and B). R392 is a conserved residue (Supplementary Figure 2) that interacts with ATP at the Hsp90-NTD nucleotide-binding pocket, critical for the stabilization of the closed conformation, and required for ATP hydrolysis [52,53]. We observed that deprotonated NY33 pulled R392 away from the ATP molecule (Figure 6B), thus destabilizing the R392–ATP interaction. Collectively, these results suggest that the nitration at Y33 may contribute to the decrease in ATPase activity we observed after peroxynitrite treatment, where oxidation reduces Hsp90 ATPase activity by 80% [27]. We also studied the behavior of the isolated Hsp90-NTD as a proxy for the dynamics of this domain in the open conformation of the homodimer. Located in this domain, the ATP-lid is a dynamic region covering amino acids 103–130 (Figure 1A). The dynamics of the ATP-lid region regulate Hsp90 ATPase activity by modulating binding to ATP and/or release of ADP [42], suggesting that changes in this structural motif may affect Hsp90β activity. In this case, we considered an open conformation of the ATP-lid region in the absence of nucleotides, as this has been described as the most stable conformation [48]. A comparative analysis of these systems dynamics showed that nitration also affected lid mobility in this conformation. The observed changes were less specific than those described for the closed dimer but showed that nitration at Y33 led to increased mobility of the lid (Sup. Figure 2), confirming that nitration of Hsp90 at Y33 affects ATP-lid dynamics.

In the case of Y56, as this residue is solvent exposed and has no direct interactions with the neighboring protomer in the Hsp90 dimer (Figure 1C), we analyzed two different systems, the isolated Hsp90-NTD and the complete Hsp90β dimer. Both simulations showed that the interaction of Y56 with several residues located in the ATP-lid can be modulated by nitration and/or the NY protonation status. Specifically, we found that ATP-lid dynamics is sensitive to Y56 nitration with very different results as a function of the NY56 acid-base behavior. NY56 in the anionic state led to repulsion of a series of ATP-lid residues (113 to 115), favoring a much more open and flexible configuration of the lid and exposing the region of NY56 to the solvent (Figure 6C). On the other hand, the hydrogen bond between the hydroxyl group of the neutral form of NY56 and A116, together with hydrophobic interactions between the nitro group and L117, stabilized a closer and rigid conformation of the lid (Figure 6D). In summary, the flexibility of the ATP-lid is significantly affected by nitration of Y56, favoring a more relaxed lid conformation when NY56 is deprotonated, and a closed conformation when NY56 is protonated. As the interaction of Hsp90 with the bound nucleotide is tightly regulated by the mobility of the ATP-lid [54], and in agreement with our functional data, the MD analysis suggests that nitration at Y33 and Y56 may affect nucleotide exchange during catalysis.

On the other hand, the simultaneous replacement of Y33 and Y56 retained the enhanced interaction of NY33 with R392 (Figure 6A–B), resulting in R392 drawing away from the ATP molecule, as well as the described effects of NY56 on ATP-lid dynamics (Figure 6C–D). Collectively, these effects on the dynamics of the ATP-lid and on the interaction of ATP with residue R392 could affect Hsp90 ATPase activity.

### Nitration at Y33 and/or Y56 differentially affects Hsp90 ATPase and chaperone holdase activities

**Figure 7:**
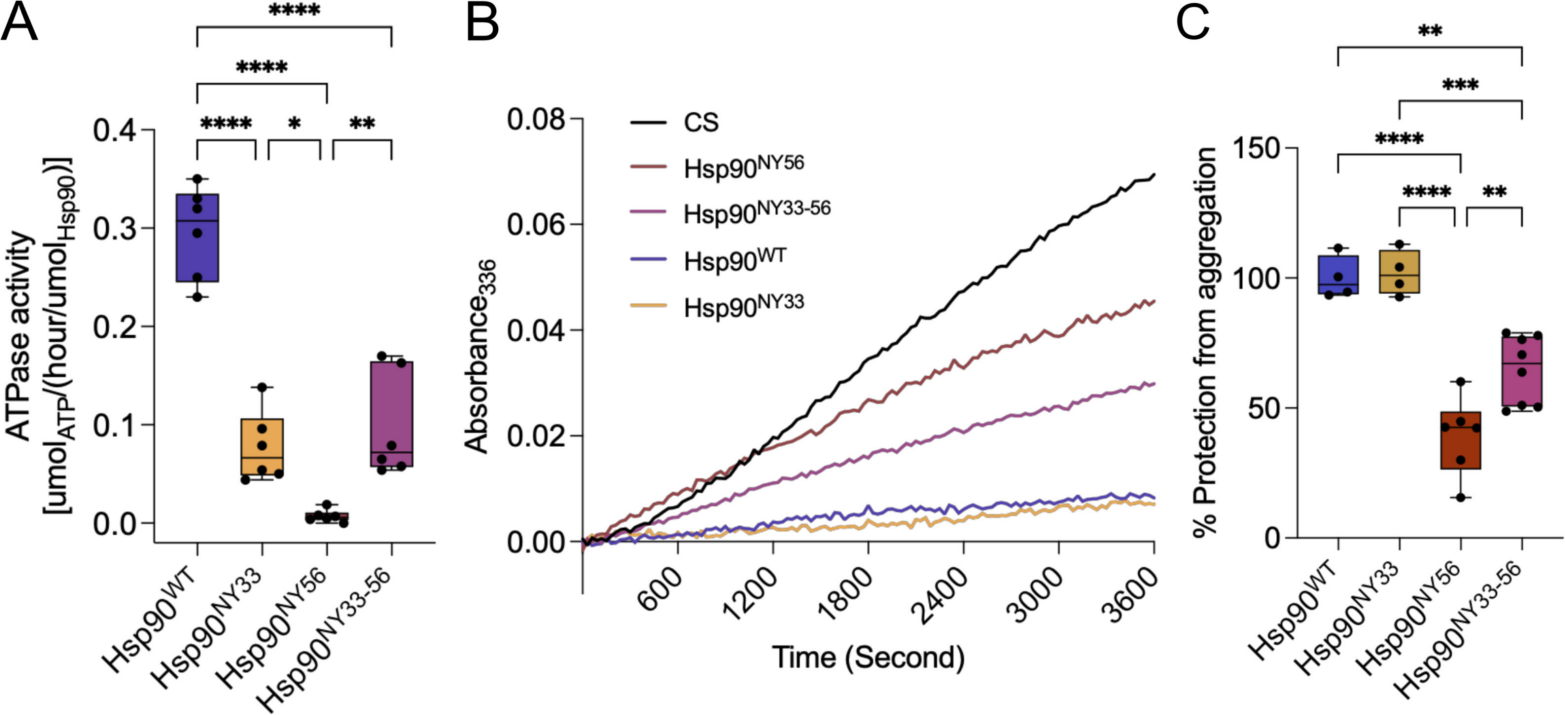
Nitration at Y33 and Y56 affects Hsp90 ATPase and chaperone holdase activities. (**A**) ATPase activity of Hsp90, Hsp90^NY33^, Hsp90^NY33^, and Hsp90^NY33-56^ expressed as μmol ATP consumed per hour *versus* Hsp90. (**B-C**) Unfolding and aggregation of citrate synthase under conditions of heat shock (42°C) in the presence or absence of Hsp90, Hsp90^NY33^, Hsp90^NY33^, and Hsp90^NY33-56^ was (**B**) followed over time by measuring absorbance at 336 nm and (**C**) expressed as percentage protection from aggregation *versus* Hsp90^WT^ (% Hsp90). Data are shown using Tukey’s representation with the median indicated in the box plot (*n* = 4–6 independent experiments). ****P<0.0001 *versus* Hsp90 by one-way ANOVA with Dunnett’s multiple comparisons *post hoc* test.

As shown in Figure 1C, both Y33 and Y56 are located in the Hsp90-NTD, near the ATP-binding pocket. The residue Y33 (Y24 in yeast) forms intra- and intermolecular interactions during Hsp90 ATP-bound state that are relevant to its ATPase activity [55] and Y56 participates in key interactions that facilitate ATP entry into the pocket and its subsequent binding and hydrolysis [52]. Thus, the structural changes induced by nitration at one or both these residues may underlie the changes in overall activity we previously described for Hsp90 after peroxynitrite treatment, accounting for a reduction of 80% in ATPase activity and 50% in chaperone holdase activity [27]. In agreement, incorporation of a single NY at position 33 decreased Hsp90 ATPase activity by 60% when compared with Hsp90^WT^ (Figure 7A), in contrast with the 80% decrease observed for Hsp90^NY56^ (Figure 7A). In agreement with a lack of additive effect on secondary and quaternary structure after simultaneous nitration at Y33 and Y56, Hsp90^NY33-56^ showed a 60% decrease in ATPase activity, similar to that of nitration at only Y33 (Figure 7A).

To investigate the effect of nitration at Y33 and Y56 on Hsp90 ATP-independent chaperone holdase activity, we evaluated the ability of Hsp90 to protect citrate synthase from unfolding and aggregation following heat shock. As we previously described [27], nitration at Y33 had no effect on Hsp90 chaperone holdase activity, as Hsp90^NY33^ prevented aggregation of citrate synthase at rates comparable with those of Hsp90^WT^, suggesting that binding to citrate synthase may stabilize the dimeric population of Hsp90^NY33^, exerting a protective effect on citrate synthase upon heat shock (Figure 7B). In contrast, Hsp90^NY56^ showed a ~50% decrease in chaperone holdase activity (Figure 7B–C), comparable with the decrease we reported previously for peroxynitrite-treated Hsp90 [25,27], suggesting that the effects of nitration at Y56 on Hsp90 structure and function are different from those observed for Hsp90^NY33^ and that although the dimeric structure was maintained, conformational changes in the dimer may be responsible for the decrease in overall activity triggered by nitration at Y56. On the other hand, and in line with previous results, the observed changes in Hsp90 structure due to simultaneous nitration at Y33 and Y56 affected the chaperone holdase activity leading to an intermediate phenotype when compared with the changes observed for Hsp90^NY33^ and Hsp90^NY56^, with a 30% reduction in activity compared with the 60% decrease observed for Hsp90^NY56^ alone (Figure 7B–C).

Together, these results suggest that nitration at either Y33 or Y56 has differential impacts on Hsp90 structure and activity and that the simultaneous nitration of Y33 and Y56 does not have an additive effect, which could define an alternative population of nitrated Hsp90, apart from Hsp90^NY33^ and Hsp90^NY56^, with distinct pathological functions.

## Discussion

A potential role for nitrated proteins as disease drivers in neurodegenerative conditions started to emerge two decades ago [56–60]. However, understanding the impact of tyrosine nitration on protein structure and function has been hindered by the lack of appropriate tools to dissect the role of a single oxidative modification when an oxidant can modify both different and multiple amino acids in a protein. Using state-of-the-art methodologies, we showed that site-specific nitration of Hsp90 on Y33 and Y56 triggers new pathological functions that cannot be compensated or overcome by unmodified Hsp90. While Hsp90 nitrated on Y56 induces motor neuron and PC12 cells [27,28], Hsp90 nitrated on Y33 induces motor neuron death but decreases mitochondrial metabolism in PC12 cells without affecting cell viability [25]. Not only do the different forms of nitrated Hsp90 have distinct functions, but they activate different signaling pathways depending on the cell type, suggesting that there is a complex interplay between the different forms of nitrated Hsp90 with distinct pathological functions [25,27,28]. Phosphorylation of Hsp90 at Y33 and Y56 has been reported in several studies [61,62]. Interestingly, although a regulatory role for Y56 phosphorylation has not been described to date, phosphorylation at Y33 (Y24 in yeast) by Wee1 regulates Hsp90 ATPase activity and ability to interact with client proteins and controls cell cycle progression in yeast [63,64]. As nitration impairs phosphorylation of a tyrosine residue and *vice versa* [23], these results suggest that the interplay between phosphorylation and nitration adds an additional layer of regulation to cellular signaling in pathological conditions. Recently, Griswold-Prenner et al. released a curated database of the nitroproteome described to date [23], highlighting the importance of tyrosine nitration in cancer biology. We showed that in schwannoma cells, nitrated Hsp90 acts as a metabolic switch to increase tumor cell proliferation. Hsp90 nitrated on Y33 decreases mitochondrial metabolism, while Hsp90 nitrated on Y56 increases glycolysis [38]. The mechanism by which site-specific nitration of Hsp90 on Y33 and Y56 elicits different new pathological functions could be linked to distinct structural changes induced by nitration on those critical residues. Here, using complementary approaches, we show that nitration of Hsp90 on Y33 and Y56 has a profound and site-specific impact on protein structure and activity, translating into new biological functions.

Hsp90 ATPase and chaperone activities are driven by the formation of a functional dimer that interacts with co-chaperones and client proteins [52,64–68]. We showed that treatment with peroxynitrite in conditions that lead to nitration on Y33, Y56, Y276, Y484, and Y596 has a significant impact on Hsp90 function, decreasing the ATPase and chaperone holdase activities by 80% and 50%, respectively [25,27]. These data suggest that oxidation, and more specifically nitration, could affect Hsp90 structure. By simultaneously replacing Y33 and Y56 by either nitration-resistant phenylalanine or NY as the sole modification in the protein, we discovered that nitration on these two critical residues induced a shift in the structure of the dimer from an open to a more compacted conformation, while inducing formation of stable oligomers corresponding to tetramers and hexamers, which could explain the changes in the ATPase and chaperone holdase activities we reported previously [25,27]. One of the oligomeric sedimentation peaks observed in the peroxynitrite-treated Hsp90 profile had a sedimentation coefficient of 11S, corresponding to tetramers, as described previously [49]. Importantly, the presence of the oligomeric species was significantly decreased after replacing Y33 and Y56 by nitration-resistant phenylalanine, with an increase in the presence of monomeric species, indicating that nitration on Y33 and Y56 contributes to Hsp90 oligomerization, but oxidation of other amino acids may also contribute to destabilizing the dimer resulting in a monomeric population of Hsp90. On the other hand, the prominent oligomeric peak in Hsp90^NY33-56^ was observed at the expected sedimentation coefficient for a hexameric population [49], suggesting that other oxidative changes in addition to Y33 and Y56 nitration may contribute to oligomerization into tetrameric species, but nitration of Y33 and Y56 is sufficient and necessary to induce formation of hexamers. This population persisted in the presence of DTT, indicating that the quaternary structure may not be mediated by disulfide bond formation or that any disulfide bond may be protected by the oligomeric arrangement (Supplementary Figure 3).

As observed for Hsp60 and Hsp100, the co-ordination of Hsp90 dimers to form oligomers could provide a scaffold for binding and efficient chaperoning of certain client proteins [49]. This reasoning is further supported by the increased association observed between Hsp90 oligomers and cochaperone Aha1 [40], and the increase in chaperone activity seen with at least two client proteins [69]. Besides enhancing interactions with existing partners, oligomerization may promote interactions with previously unidentified clients, thus expanding the interactome of Hsp90, leading to the gain-of-function we have previously observed in schwannomas and neuronal cells [25–28]. While multiple studies have detected the presence of Hsp90 oligomers [40,46,49,70–74], the basis of this quaternary structure change is still unclear but could be driven by selective tyrosine nitration. *E. coli* HtpG bound to ADP was found to crystallize as a tetramer, described as a ‘dimer of dimers’, with one dimeric unit inverted over the other [46]. The formation of this structure is dependent on the Hsp90-NTDs being in the closed conformation, bound to nucleotides. In this closed state, the Hsp90-NTDs interact with the Hsp90-MD and Hsp90-CTD of the other dimer, shielding exposed hydrophobic residues across the interacting domains. Notably, the Hsp90^NY33-56^ dimer presented as a closed dimer conformation in the SV-AUC data, which could promote oligomer formation, resulting in the stable hexamer peak also observed in the sedimentation profile. Multiple studies have discussed the possibility of NTD interaction across dimers mediating oligomer formation [40,73]. Divalent cation-based or heat-based oligomerization of Hsp90 leads to formation of tetramers and hexamers [49,69]. Tetramers induced by Mg^2+^ seem to be less stable than the hexameric species, which may explain the small tetrameric population present after peroxynitrite treatment. In the presence of Mg^2+^, Hsp90 hexamer adopts a clustered ‘cozy nest’ structure, with the dimer as the building unit [40,49]. We detected the nitration-induced oligomers in native gels after 48 h incubation at room temperature in the absence of cross-linking, suggesting they are more stable than heat/cation-induced oligomers. The tyrosine nitration-induced changes in structure also affected Hsp90 function. Both Y33 and Y56 are located in close proximity to the ATP-binding pocket in Hsp90-NTD, suggesting that nitration of these residues could affect Hsp90 intrinsic ATPase and chaperone holdase activities. In agreement, the simultaneous incorporation of NY in positions 33 and 56 significantly reduced the ATPase activity by ~60%, and the chaperone holdase activity by ~30%, suggesting that oxidative modifications in other amino acids, such as Y276, Y484, and Y596 that we showed are also prone to nitration, may contribute to the decrease in ATPase and holdase activity observed in the oxidized protein after peroxynitrite treatment [25,27].

**Figure 8:**
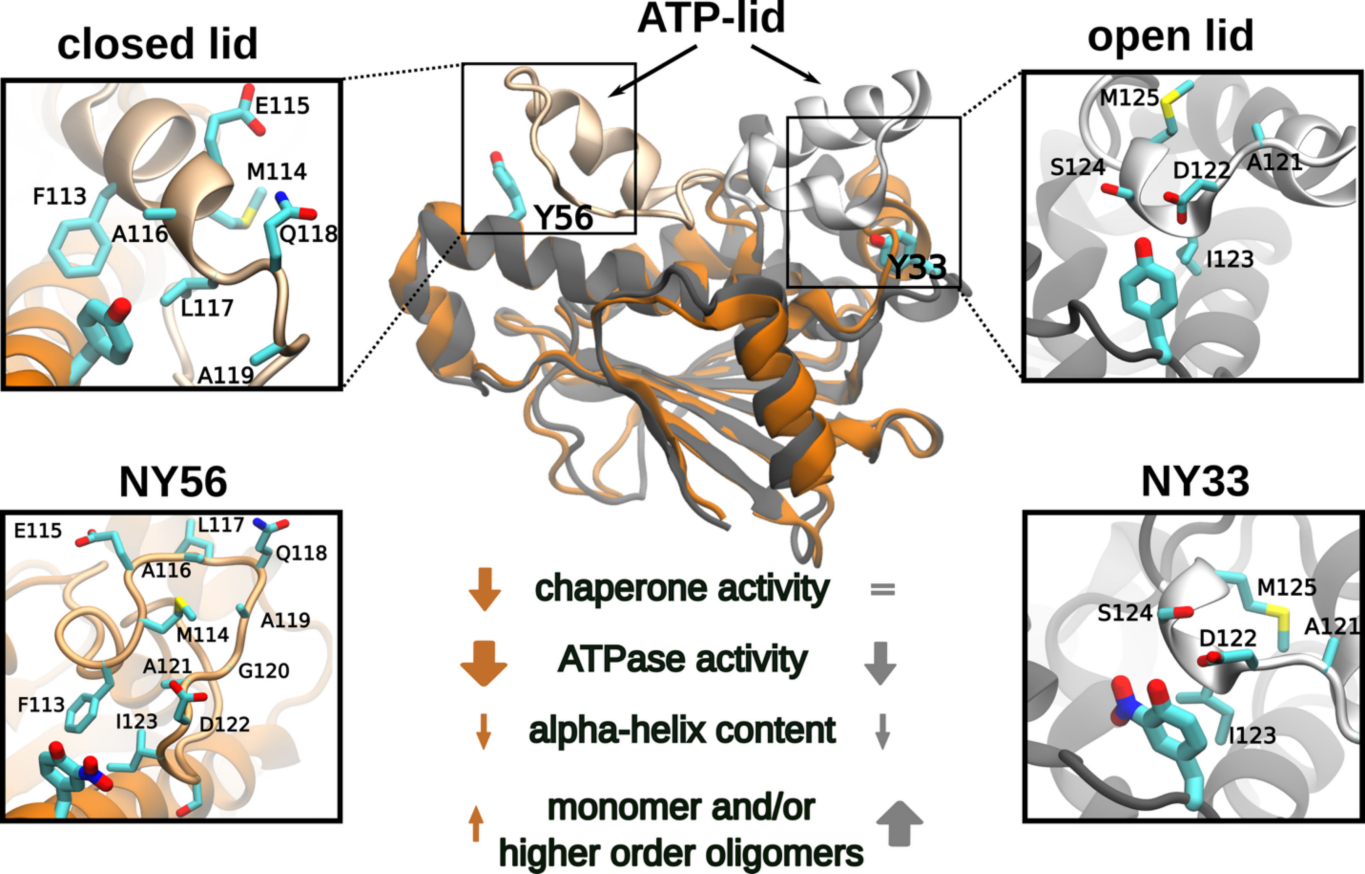
Schematic representation of the structural and functional effects observed for both Y33 or Y56 nitration. Nitration of Hsp90 at Y33, with the nitrotyrosine in the deprotonated state, pulls the R392 away from the ATP molecule. Nitration of Y56 affects the ATP-lid dynamics, with opposite effects depending on the protonation state. A deprotonated nitrotyrosine leads to a more open and flexible conformation, while the protonated form leads to a more rigid ATP-lid. Bottom, summary of the structural and functional changes triggered by nitration at Y33 and Y56.

In different cell culture models, including motor neurons, PC12 cells, and schwannoma cells, we showed that approximately 1–15% of Hsp90 is nitrated on Y33 and/or Y56, depending on the cell type, and that nitration on Y33 triggers different biological functions as compared with Hsp90 nitrated on Y56. Further, in schwannoma cells, Hsp90 endogenously nitrated on Y33 (Hsp90^NY33^) is localized in the mitochondria and cytosol, while Hsp90 endogenously nitrated on Y56 (Hsp90^NY56^) also localizes in the nucleus [26,38]. In three-dimensional (3D) schwannoma cell culture models, Hsp90^NY33^ is present in cells in the periphery of the cell aggregates, while Hsp90^NY56^ is distributed throughout the 3D structure, suggesting that Hsp90^NY33^ and Hsp90^NY56^ may confer distinct functional advantages to cells in different locations within a tumor [38]. Collectively, these data suggest that these two forms of nitrated Hsp90 undergo specific and distinct structural changes that trigger different pathological functions. Interestingly, using AUC, we observed a prominent hexameric, stable population in Hsp90^NY33^ and a dimer in rapid exchange with monomeric species, suggesting that the dimer may lose stability in solution but could be stabilized upon binding to client proteins and/or co-chaperones and by forming oligomers, as suggested by the chaperone holdase assays in which Hsp90^NY33^ afforded citrate synthase complete protection from aggregation during heat shock, as we had previously shown [25]. It is also possible that the oligomers may retain chaperone holdase activity and be responsible for the new function. In contrast, Hsp90^NY56^ was mainly in a stable dimeric form, with a minor oligomeric population. However, the significantly decreased ATPase and chaperone holdase activities in Hsp90^NY56^ suggest the presence of an altered dimer conformation compared with wildtype Hsp90. These significant structural differences between Hsp90^NY33^ and Hsp90^NY56^ may explain their distinct pathological activities. In agreement, nitration on Y33 and/or Y56 differentially affected Hsp90 ATPase activity. For example, while NY56 almost abolished Hsp90 ATP hydrolysis, NY33 reduced it by 60%. At the molecular level, ATP binding and subsequent hydrolysis by Hsp90 is co-ordinated by interactions between the Hsp90-NTD and Hsp90-MD that span both protomers of the Hsp90 dimer [29,52,65]. A crucial residue in these interactions is R392, located in a loop in the Hsp90-MD that neighbors the Hsp90-NTD in the closed conformation. Previous studies have established the importance of R392 in stabilizing the Hsp90 closed conformation via a polar interaction with the gamma phosphate of ATP [53], while other residues in the Hsp90-MD loop interact with Y33 in the NTD of the other protomer [52]. The *in silico* data showed a change in this interdomain interface upon nitration of Y33. In the presence of NY33, the close interaction between ATP and R392 was perturbed, and the increased distance (~7 Å to ~14 Å) between these interacting groups would destabilize the closed ATP-bound conformation of the protein. This is similar to the effects seen when R392 is mutated to a less polar residue and can no longer bond with ATP, resulting in significant loss of ATPase activity [65]. The lowered rate of ATP hydrolysis observed in Hsp90^NY33^ can therefore be explained by the loss of stability evident in the ATP-bound conformation. Interestingly, R392 and Y33 are almost strictly conserved, suggesting that both residues are critical to Hsp90 function (Supplementary Figure 4). In contrast with NY33, by performing MD simulations, we observed that the increased interactions between NY56 and residues located in the ATP-lid affected the flexibility of the lid. We observed that the increased interaction between NY56 in the protonated state and residues such as Q118 and A119 reduced the flexibility of the ATP-lid, while NY56 in the deprotonated state had the opposite effect. Closure of the lid after ATP binding drives dimerization of the Hsp90-NTDs, forming the closed and active Hsp90 conformation [52]. Reduced lid flexibility would affect the entry of ATP into its binding pocket as well as subsequent lid closure and Hsp90-NTD dimerization. The ATP-lid dynamics not only modulate nucleotide-binding but also the dynamics of opening and closing of the full-length dimer. Thus, besides the effect on ATP hydrolysis, nitration at Y56 could affect other activities of Hsp90, including its ability to effectively bind clients [75], which could explain the 50% decrease observed in Hsp90^NY56^ chaperone holdase activity. The changes in Hsp90 intermolecular interactions and in the ATPase and holdase activities upon nitration at Y33 or Y56 are summarized in Figure 8.

The absence of any direct cross-interactions between the two NY residues when they are simultaneously present is unsurprising considering the significant distance between them (~28 Å). This separation also suggests that the simultaneous nitration at Y33 and Y56 may have an additive effect on overall Hsp90 functionality. Surprisingly, nitration at Y56 resulted in 80% reduction in ATPase activity, compared with the 60% decrease observed after nitration of only Y33 or both, Y33 and Y56. Similarly, nitration at Y56 led to a 50% decrease in chaperone holdase activity *versus* a 30% loss caused by nitration at Y33 and Y56, and, as we previously showed, nitration at Y33 had no effect on chaperone holdase activity [25]. Thus, nitration at Y56 had the most impactful effect on both the holdase and ATPase activities, while the presence of both nitrated residues attenuated this effect. The absence of a monomeric peak for Hsp90^NY33-56^ suggests that the simultaneous nitration of both residues does not destabilize the dimer but leads to formation of stable hexameric species and a more compacted dimer conformation. It is possible that NY33 exerts a stabilizing or attenuating effect over NY56 when present together. Interestingly, nitration at Y56 facilitated nitration at Y33, suggesting that Hsp90 nitrated at Y56 may originate a small fraction of the chaperone that is nitrated at both residues and, similar to Hsp90^NY33^ and Hsp90^NY56^, also carries proliferative activity and could regulate protein location. Whether this subpopulation could be present in tumor cells in basal conditions or appear in conditions of increased peroxynitrite production warrants future studies. In terms of Hsp90 intrinsic activities, as shown here, the behavior of the nitrated residues is largely dependent on, firstly, the protonation state of the nitro group, and secondly, on the open or closed state of the ATP-lid. Given the notoriously transient conformations of the ATP-lid in human Hsp90 in solution [76], as well as the lack of predictability associated with determining the protonation state of an NY residue, it is expected that this protein exists in a mixture of conformational states when in solution, and the resulting interplay gives rise to the effects observed in the *in vitro* assays and could alter the interaction of Hsp90 with cellular partners. Previous studies have discussed oligomerization as a protective mechanism in Hsp90, allowing it to retain its functionality under conditions of stress [70]. The stressful environments described in the literature are limited to either conditions of high heat (above 49°C) or excessive presence of divalent cations (Ca^2+^ and Mg^2+^). We propose an additional possible scenario where oxidative and nitrative stress in pathological conditions may cause nitration and, depending on the nitration site, subsequent oligomerization of Hsp90, with the oligomers retaining functionality. Thus, the Hsp90 dimer with an altered conformation triggered by nitration on Y56, and the oligomers formed by nitration on Y33 hold the potential for altered associations with co-chaperones [66] and client proteins, redefining the Hsp90 interactome [70], and leading to the observed pathological functions of nitrated Hsp90 in disease states with underlying chronic inflammation.

In the last 25 years, growing evidence has supported Hsp90 as an exceptional drug target for the treatment of neurodegenerative diseases and cancer, but the associated toxicity has hindered the success of its inhibitors [36]. A comprehensive understanding of the functional and structural changes induced by nitration in Hsp90 could lead to the development of new therapeutic approaches that selectively target the nitrated forms of Hsp90 and inhibit their pathological function. Leveraging the modified structure of nitrated Hsp90 could spare Hsp90 normal functions, yielding therapeutic strategies with minimal side effects.

## Materials and methods

### Hsp90 expression and purification, and treatment with peroxynitrite

Human Hsp90β was expressed and purified as we described [27], with modifications. Briefly, the sequence of human Hsp90β (Hsp90^WT^) optimized for bacterial expression (GenScript, U.S.A.) was cloned in a pBad/Myc-Hisx6 vector (Invitrogen). The codons encoding for Y33 and Y56 were either replaced by a codon encoding for phenylalanine (Genscript) or by a TAG stop codon. The incorporation of an NY by genetic code expansion into the selected position was performed using a newly selected orthogonal NYe-bearing suppressor tRNA and engineered tRNA synthetase pair (pDule-nitroTyr-A7) [77]. Each pBad-Hsp90^NY^ (TAG) plasmid was co-transformed with pDule-nitroTyr-A7 [77] into *E. coli* BL21-AI cells. Hsp90^WT^ and Hsp90^F33-F56^ were expressed in BL21-AI cells grown in LB and induced at OD 0.8 with 0.2% arabinose at 37°C and 220 rpm for 6 h. The Hsp90^NY^ proteins produced by genetic code expansion were expressed in autoinduction medium containing 1 mM NY for 28 h [78]. All Hsp90 recombinant proteins were purified using the following scheme: Cells were pelleted at 4500×g, resuspended in 20 mM lysis buffer (20 mM phosphate buffer pH 8, 100 mM PMSF, 10 U/L DNAse and 0.5 mM lysozyme) and incubated for 30 min at 4°C. The lysate was homogenized using 3–5 rounds of sonication at 15–20 watts for 90 s intervals. Cell debris was pelleted in Oakridge tubes at 18000×g for 40 min at 4°C. A 20 ml supernatant was filtered through a 0.45 µm Supor membrane and loaded onto a Ni-NTA resin at 3 ml/min, washed with 80 ml wash buffer, and eluted with 50 ml of a 0–100% gradient of elution buffer (20 mM sodium phosphate, 100 mM NaCl, 400 mM imidazole, pH 8). All Hsp90 constructs eluted at 200–350 mM imidazole concentration. Pure elution fractions for each protein were combined, concentrated, and loaded onto a 16/92 Sephacryl-300 column for size exclusion chromatography. The column was washed, and protein was eluted using PBS at pH 7.4. Protein was concentrated using a vacuum concentrator setup and used for experiments immediately or stored at 1 mg/ml in PBS pH 7.4 at −80°C. Peroxynitrite (~140 mM in 1 M NaOH) was diluted 1:15 in mqH_2_O and quickly added to 1 mg/ml (11.6 μM monomer) of either Hsp90^WT^ or Hsp90^F33-F56^ to a final concentration of 0.5 mM with constant agitation, as we previously described [27]. For the slot blots, Hsp90^WT^, Hsp90^NY33^, and Hsp90^NY56^ treated in the same conditions described above with increasing concentrations of peroxynitrite, as indicated.

### Circular dichroism

Far-UV CD spectra were collected on a JASCO 720 spectrophotometer, at path length 1 mm, bandwidth 1.0 nm and protein concentrations of 2–3 μM. Samples were dialyzed into sodium phosphate buffer pH 7.5 prior to the experiment. An average of three scans was collected to generate the final spectrum and corrected using the signal from the experimental buffer. Secondary structure composition of each sample was analyzed using BeStSel [79].

### Native PAGE and stability assays

To study nitration-induced changes in Hsp90 native conformation, 20 μg of Hsp90^WT^ or the different site-specific nitrated forms of Hsp90 (1 mg/ml) were loaded onto 4–20% polyacrylamide gels (Bio-Rad) in native loading buffer (Tris pH 6.8, 50% glycerol, 0.2% bromophenol blue). Native PAGE ladder (Thermo) and 1 mg/ml BSA were used as standards for all native PAGE gels. For the stability assays, proteins were incubated at 25°C and 4°C in PBS (pH 7.4) for 0 h, 12 h, and 48 h after purification, followed by native PAGE, as described.

### Intracellular protein delivery and cell growth assays

Human recombinant Hsp90^WT^, Hsp90^WT^ treated with 0.5 mM peroxynitrite, Hsp90^NY33^, Hsp90^NY56^, and Hsp90^NY33-56^ were intracellularly delivered into human Schwann cells or mouse schwannoma cells using the permeant agent Chariot as we previously described [25,27,38]. Briefly, a 200 μl mixture containing 10 μg of recombinant protein in 100 μl DPBS and 4 μl of Chariot (Active Motif, Carlsbad, CA, U.S.A.) in 100 μl ddH_2_O was incubated at room temperature for 30 min. A pellet containing 1 × 10^6^ schwannoma or Schwann cells was resuspended in the protein/Chariot mixture, followed immediately by the addition of 400 μl DMEM. Cells were incubated for 1 h at 37°C, 5% CO_2_. The cells were gently resuspended at 20 min intervals. After the last resuspension, 1.0 ml Schwann cell culture media containing 16% fetal bovine serum was added, and cells were incubated by an additional 1 h. The cells were then seeded at a density of 10,000 cells per well in a 96 well CellBind plate (Cat. no. 3300, Corning) and incubated for either 24 h (human Schwann cells) or 48 h (schwannoma cells). Cells were then rinsed once with PBS and incubated with 50 µl 0.2% crystal violet (Cat. no. F906-03, J.T.Baker, NJ, U.S.A.) in 20% methanol per well for at least 20 min. Wells were rinsed with ice-cold distilled water and the crystal violet solubilized with 200 µl of 1% SDS per well. Absorbance at 595 nm was measured for each well (SpectraMAX Plus, Molecular Devices, CA, U.S.A.). Cell growth is expressed as a percentage of Hsp90^WT^.

### Quantitative infrared slot blots

The slot blots were performed as previously described [27,38]. Briefly, 0.5 μl of the recombinant proteins -/+ peroxynitrite treatment were loaded directly onto a precut 0.45 μm Immobilon-FL PVDF membrane (Cat. no. IPFL00010, Millipore) placed in the slot blot apparatus (Model no. DHM-96, Scie-Plas, United Kingdom). The nitrocellulose membrane was rinsed three times and then blocked using TBS Odyssey Blocking Buffer (Cat. no. 927–60001, Li-Cor Biosciences, Lincoln, NE, U.S.A.) and incubated overnight at 4°C with their indicated in-house developed primary antibodies against nitrated Hsp90, or an anti-myc tag antibody [25,27,38]. IRDdye secondary goat antibodies (Li-Cor Biosciences), anti-mouse (680RD, Cat. no. 925–68070), and anti-rabbit (800CW, Cat. no. 925–32211) were used at a 1:20,000 dilution. All slot blots were visualized and quantified with the Odyssey System (Li-Cor Biosciences). Standard curves of site-specifically nitrated Hsp90, either Hsp90^NY33^ or Hsp90^NY56^, were included.

### ATP hydrolysis assay

To assess the geldanamycin-sensitive Hsp90 ATPase activity, Hsp90^WT^ (10 uM) was incubated with ATP (2 mM) in the presence or absence of the Hsp90 inhibitor geldanamycin (4 uM) for 3 h at 37°C, and ATP hydrolysis was calculated by determining inorganic phosphate production using the Enzchek phosphate detection kit (Thermo Scientific), as previously described [37]. A Shimadzu UV-VIS 2401 spectrophotometer was used to detect phosphate production at 360 nm, and the absorbance was extrapolated to phosphate concentration using a standard curve.

### Citrate synthase aggregation assay

Hsp90 chaperone holdase activity was assessed as previously described [25,27] . Briefly, citrate synthase (5 μg) was incubated in 40 mM HEPES at 42°C, and enzyme inactivation and aggregation were followed in the presence or absence of 10 μg of Hsp90^WT^ or the different forms of nitrated Hsp90 at 336 nm over 60 min, using a Shimadzu UV-VIS 2401 spectrophotometer. The chaperone holdase activity was expressed as percentage protection from aggregation *versus* Hsp90^WT^.

### Sedimentation velocity-analytical ultracentrifugation (SV-AUC)

SV-AUC was performed using a Beckman Coulter Optima XL-A analytical ultracentrifuge. Two-channel sectored cells with 12 mm pathlength were used for all samples. Samples were spun at 42,000 rpm at 20°C for 300 scans, acquired at 280 nm absorbance. Concentration of each protein was at 11–15 μM, and prior to the experiment, Hsp90^WT^ + ONOO^-^ and Hsp90^F33-F56^ + ONOO^-^ were dialyzed post-peroxynitrite treatment to prevent buffer mismatch. Data were fit to the continuous c(S) distribution using SEDFIT [80]. Buffer density and buffer viscosity were estimated using SEDNTERP [81].

### Computational modeling

Two different protein systems were considered and subjected to MDs for the initial model generation: the isolated N-terminal domain (Hsp90-NTD), and the entire homodimer of human Hsp90β. For the Hsp90-NTD system, the initial model was generated from the crystallographic structure in which the ATP-lid is in the open conformation (PDBid 5UCJ), and after removing the inhibitor present in the structure. In the case of systems with ATP at the active site and the ATP-lid in the closed conformation, the crystallographic structure in the presence of ATP (PDBid 3T0Z) was used to build the initial model. To study the dynamics of Hsp90β homodimer, the initial model was created using three different strategies due to the lack of an experimentally determined complete structure. Two different templates were subjected to homology modeling [82], the structure of the human Hsp90-Cdc37-Cdk4 complex (PDBid 5FWK), and the Hsp90β structure from yeast (PDBid 6XLC). The third model was generated using AlphaFold 2 [83,84]. Since the dynamic behavior of these three models showed no significant differences, the complete analysis was performed using PDBid 5FWK. In all cases, the dynamic properties of these systems were evaluated and compared in the presence or absence of nitrated Y33 and/or Y56 and considering both possible protonation states. For the MDs, each of the described systems was analyzed in explicit solvent following standard protocols. Briefly, the systems were immersed in an octahedral TIP3P water box [85], using a minimum distance of 12 Å to the box edge in the case of the Hsp90-NTD systems, or 10 Å for the homodimers. Protein parameters were generated using the Amber ff14SB force field [86], while parameters for ATP and ADP [87] or 3-NY, both in protonated and deprotonated states, were as previously described [88]. Every simulation was performed with AMBER 18 [89] under periodic boundary conditions with a 10 Å cutoff for the calculation of nonbonded interactions. Hydrogen bond lengths were maintained at their equilibrium distance using the SHAKE algorithm, while temperature and pressure were kept constant using a Langevin thermostat and an isothermal-isobaric barostat (NPT). First, systems underwent a minimization process of 100–1000 allowed steps until convergence was reached, and then an equilibration and thermalization protocol was performed to heat systems to 300 K over 25 ps. Finally, production MDs were performed. Dynamics visualization and molecular drawings were performed with VMD [90], and trajectory analysis was done using the cpptraj module of Ambertools [89].

### Statistical analysis

Statistical analysis, including testing for outliers and normal distribution, was performed using GraphPad Prism (GraphPad Software Inc., San Diego, CA, U.S.A.). The ATPase and chaperone holdase activity of Hsp90 and the different site-specific nitrated forms of Hsp90 were assessed either simultaneously (ATPase activity) or in tandem (citrate synthase aggregation assay). Thus, more than two groups were considered for the statistical analyses. One-way ANOVA with Dunnett’s multiple comparisons *post hoc* tests was used, as the populations followed a normal distribution. The data were visualized using Tukey’s box plots, with the median indicated. Statistical significance was set at <0.05.

## Supplementary material

Supplementary Information

## Data Availability

The original contributions presented in the study are included in the article/supplementary material. Further inquiries can be directed to the corresponding author. Summarized versions of each MD simulation analyzed in this work are available for download at: https://redata.anii.org.uy/dataset.xhtml?persistentId=doi:10.60895/redata/YLVSF0.

## Open Access

This article has been published open access under our Subscribe to Open programme, made possible through the support of our subscribing institutions, learn more here: https://portlandpress.com/pages/open_access_options_and_prices#conditional

## Abbreviations

ATP: adenosine 5'- triphosphate
AUC: analytical ultracentrifugation
CD: circular dichroism
C-terminal: carboxy terminal
DTT: dithiothreitol
HEPES: N-2-hydroxyethylpiperazine-N-2-ethane sulfonic acid
Hsp90: heat shock protein 90
Hsp90-MD: Hsp90 middle domain
Hsp90-NTD: Hsp90 N-terminal domain
MD: molecular dynamics
NY: nitrotyrosine
Ni-NTA: nickel^2+^ ion coupled to nitrilotriacetic acid
N-terminal: amino terminal
ONOO^-^: peroxynitrite
PAGE: polyacrylamide gel electrophoresis
PBS: phosphate-buffered saline
PMSF: phenylmethylsulfonyl fluoride
P2X7R: P2X7 receptor
RNS: reactive nitrogen species
ROS: reactive oxygen species
S: sedimentation coefficient
SV: sedimentation velocity
