# Supplementary material for: Nitration-driven structural changes in Hsp90 linked to gain of pathological functions
Source: Biochem J. 2025 Aug 20;482(16):1209–28. doi: 10.1042/BCJ20253230 (PMC12599251; doi:10.1042/BCJ20253230)
Supplement: Supplementary Information [file bcj-482-16-BCJ20253230-s001.pdf]

Supplementary Information

Nitration-driven structural changes in Hsp90 linked to gain of pathological functions

Tilottama Chatterjee[1], Alfonso Taboada[2,3,4], Isabelle E. Logan[1,5], Patience N. Paul[5], Miranda Huerta[1], Patrick Reardon[1], Rafael Radi[3,4], Ari Zeida[3,4], Maria Clara Franco[1,5,6 #]

[1] Department of Biochemistry and Biophysics, Oregon State University, Corvallis, OR 97331, USA.
[2] Departamento de Métodos Cuantitativos, Facultad de Medicina, Universidad de la República, Gral Flores 2125, CP 11800 Montevideo, Uruguay.
[3] Departamento de Bioquímica, Facultad de Medicina, Universidad de la República, Avda. Gral Flores 2125, CP 11800 Montevideo, Uruguay.
[4] Centro de Investigaciones Biomédicas (CEINBIO), Facultad de Medicina, Universidad de la República, Avda. Gral Flores 2125, CP 11800 Montevideo, Uruguay.
[5] Center for Translational Science, Florida International University, 11350 SW Village Parkway, Port St. Lucie, FL 34987, USA.
[6] Department of Cellular and Molecular Medicine, Herbert Wertheim College of Medicine, Florida International University, Miami, FL, 33199, USA.

[#] Corresponding author
Email: marfranc@fiu.edu
Address: 11350 SW Village Pkwy, Port St. Lucie, FL 34987

Running title: Nitration alters Hsp90 structure and function

Keywords: reactive nitrogen species (RNS), tyrosine nitration, heat shock protein 90 (Hsp90), post-translational modification (PTM), protein structure, peroxynitrite, structure-function, free radicals

This file includes:

Supplementary Figures 1 to 4.

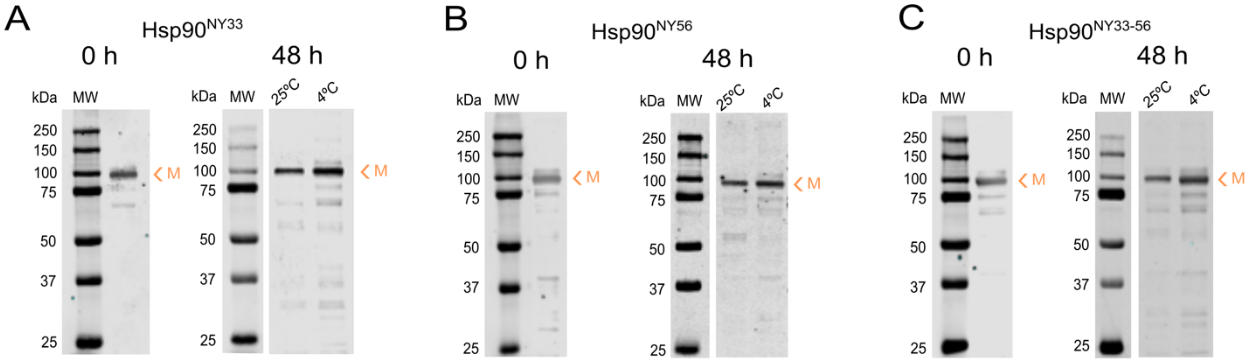

Supplementary Figure 1. The oligomeric species present in the site-specific nitrated proteins disassemble in denaturing conditions. (**A**) Hsp90^{NY33}, (**B**) Hsp90^{NY56}, and (**C**) Hsp90$^{NY33-56}$ were incubated or not at 4°C or 25°C for 48 h before denaturing, followed by infrared western blot blotted with an anti-myc-tag antibody. M: monomer.

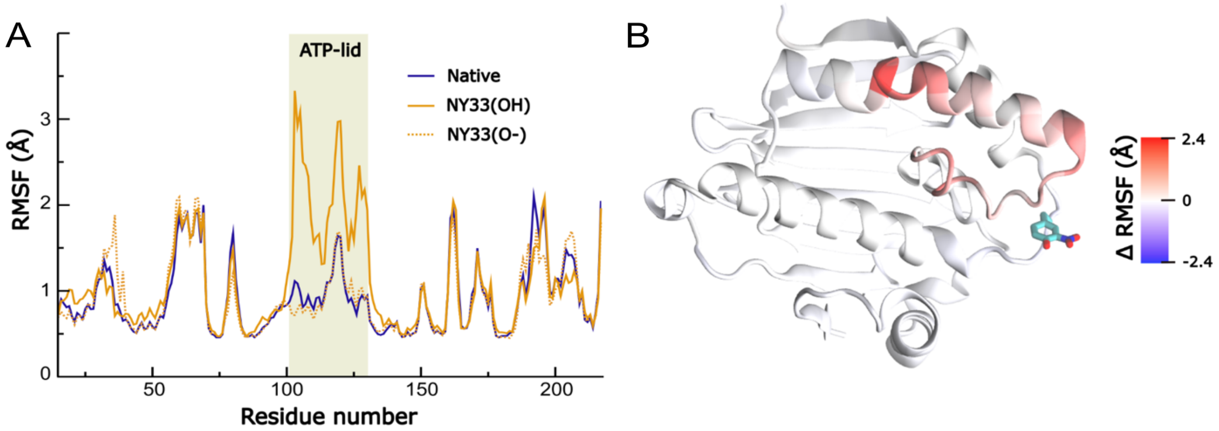

Supplementary Figure 2. Intramolecular interactions of NY33 with the ATP lid region. (A) Root mean square fluctuations (RMSF, Å) of the NTD residues as a function of Y33 state, highlighting the ATP-lid region. (B) The RMSF difference between NY33(O$^-$) and native (*up*) or protonated -NY33(OH)- and native (*down*) are mapped on the NTD structure.

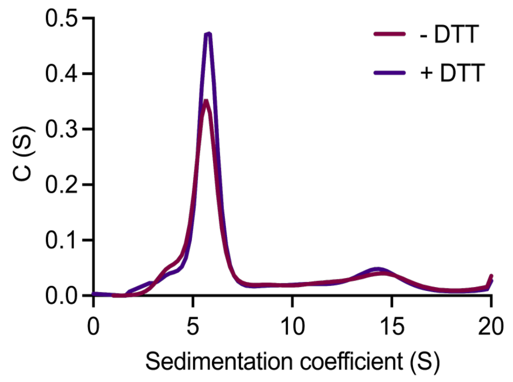

Supplementary Figure 3. The oligomeric species of Hsp90$^{NY33-56}$ are stable in the presence of a reducing agent. Sedimentation profile of Hsp90$^{NY33-56}$ with and without the addition of 1 mM dithiothreitol (DTT).

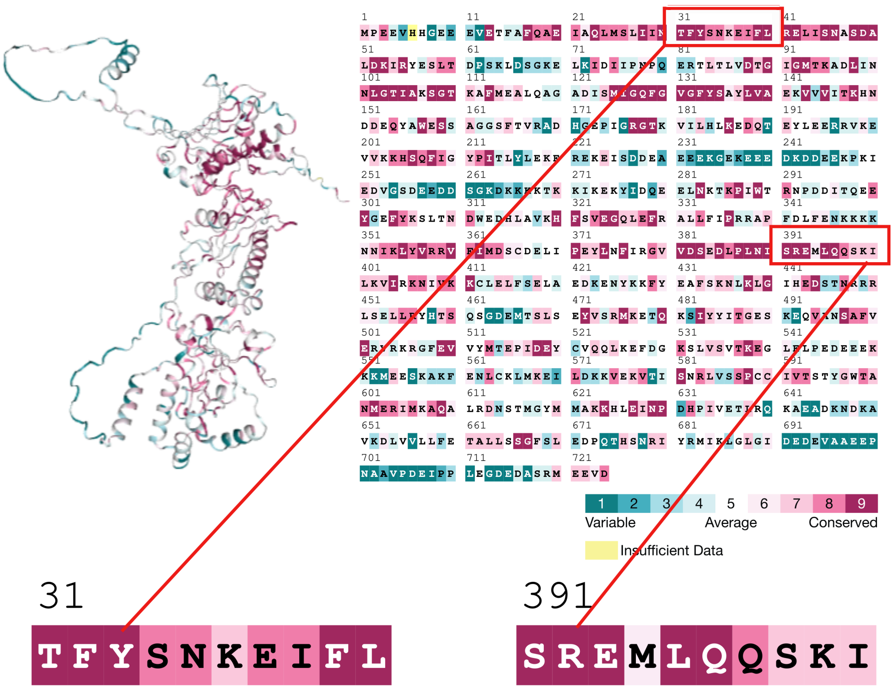

Supplementary Figure 4. Strong conservation of Y33 and R392 in Hsp90 sequence. Sequence conservation analysis of Hsp90 shows strong conservation of both Y33 and R392.
